# ZAP-X: A Novel Radiosurgical Device for the Treatment of Trigeminal Neuralgia

**DOI:** 10.7759/cureus.8324

**Published:** 2020-05-27

**Authors:** Pantaleo Romanelli, Cynthia Chuang, Antonio Meola, Radhika M Bodduluri, John R. Adler

**Affiliations:** 1 Neurosurgery, Cyberknife Center, Centro Diagnostico Italiano, Milano, ITA; 2 Department of Radiation Oncology – Radiation Physics, Stanford University School of Medicine, Palo Alto, USA; 3 Department of Neurosurgery, Stanford University School of Medicine, Palo Alto, USA; 4 Engineering, Zap Surgical Systems, San Carlos, USA; 5 Department of Neurosurgery, Stanford University School of Medicine, Stanford, USA; 6 Radiation Oncology, Stanford University Medical Center, Stanford, USA

**Keywords:** stereotactic radiosurgery, radiosurgery, srs, trigeminal neuralgia, neurosurgery, radiation oncology, zap, zap-x, cyberknife, gamma knife

## Abstract

Introduction

The treatment of trigeminal neuralgia (TN) is one of the most demanding of all radiosurgery procedures, requiring accurate delivery and sharp dose fall off. ZAP-X®, a new, innovative frameless radiosurgical device, maybe an attractive platform for the treatment of TN and other functional brain disorders. Here, we compared the dosimetry of ZAP-X plans for a single patient to that generated by a well-established dedicated radiosurgery device, the CyberKnife.

Methods

Radiosurgery plans that delineated the cranial nerve from a single patient’s fused computed tomography and magnetic resonance imaging (CT-MR) data set were planned on both the ZAP-X and CyberKnife, with the latter serving as a validated benchmark. The same target and treatment planning constraints were applied. Plans were evaluated by a physician with experience treating TN and a medical physicist. The ZAP-X treatment plan used two isocenters delivered through 4-mm collimators based on a non-isocentric plan that delivered 29,441 MU through 81 beams. The CyberKnife plans used a 5-mm collimator for a non-isocentric plan that delivered 17,880 MU through 88 beams.

Results

Based on visual inspection, the isodose volumes covered by ZAP-X and CyberKnife were similar at the prescription isodose (70% and 80%, respectively, with a maximum dose (Dmax) of 7500 cGy. The conformality index was better for the CyberKnife as compared to ZAP-X. However, the irradiated volumes were smaller at the 50%, 20%, and 10% isodoses for ZAP-X (0.12 cc, 0.57 cc, and 1.69 for ZAP-X; 0.18 cc, 0.91 cc, and 3.41 cc for CyberKnife). In particular, the 20% and 10% isodose volumes were much smaller for ZAP-X, especially on the axial and sagittal planes.

Conclusions

ZAP-X treatment planning for TN compares favorably with equivalent planning on CyberKnife. The brain volumes containing the 20% and 10% isodoses are smaller using ZAP-X, thus relatively sparing critical structures close to the target, including the Gasserian ganglion and brainstem. This feature could be of clinical relevance by potentially reducing treatment-related complications.

## Introduction

ZAP-X® (ZAP Surgical Systems, Inc., San Carlos, CA) is a new, image-guided medical linear accelerator (LINAC)-based radiosurgery system dedicated to the treatment of intracranial and cervical spine lesions. ZAP-X is characterized by a 3 megavolt (MV) S-band LINAC with a focal spot size of 2 mm, a dose rate of 1,500 MU/min at the isocenter, with dose linearity of 3%, a beam penumbra of 1.8 mm using the 4-mm collimator, and beam symmetry of less than 2% [1-2]. The ZAP-X linac is mounted within a combination of yoked gimbals, each of which accurately rotates around a common isocenter, thus allowing >2π steradian solid angle beam coverage. ZAP-X is “self-shielded” in that nearly all radiation is contained within the device, allowing it to be used safely without a radiation therapy vault; under standard operating conditions, at 1 m distance from the perimeter of the device, the maximum expected radiation equivalent dose level is below 1 mSv/year, which is equivalent to the limit for public exposure [2].

Following extensive testing, the ZAP-X system was found to meet safety, accuracy, and performance requirements widely accepted in the radiation oncology and radiosurgery industry [1-2]. Due to a variety of factors, including the lower beam energy (3 MV versus the 6 MV typical of other radiosurgical LINAC systems), the limited penumbra, the availability of a 4-mm collimator, and the ability to perform non-isocentric planning, ZAP-X also provides an interesting platform for functional radiosurgery treatments [1,3-4].

Radiosurgical treatment of trigeminal neuralgia (TN) is the most common procedure in functional radiosurgery. Such treatments are characterized by the delivery of high doses (60 to 120 Gy) to a focal central nervous system (CNS) target in order to modulate pain or movement disorders. A systematic review of the literature focused on the outcomes of stereotactic radiosurgery (SRS) for TN found 65 studies reporting on 6,461 patients [5]. While most of the reported cases were treated with frame-based SRS, frameless (mask-based), image-guided SRS using conventional linear accelerators and CyberKnife (Accuray Incorporated, Sunnyvale, CA) have also been demonstrated as safe, effective, and less invasive treatment options for TN [6-8]. Consequently, a comparison of the dosimetric quality of the frameless ZAP-X and CyberKnife plans for TN would represent an important first step in determining the suitability of ZAP-X for high-dose functional treatment. The objective of this paper is to document such a preliminary comparison.

## Materials and methods

To evaluate the ability of ZAP-X to deliver TN treatments, we developed SRS treatment plans for the frameless ZAP-X and CyberKnife systems; the latter used as a “gold standard” benchmark. Treatment plans were developed using the same CT-MR set, and the same target and treatment planning constraints were applied. The target covered a 6-mm segment of the trigeminal nerve in the retrogasserian location [7-8]. The trigeminal nerve volume receiving the prescribed dose was the same (0.03 cc). Both plans were developed and evaluated by independent physician and physicist teams (one team for ZAP-X and one team for CyberKnife), even though both teams used the same imaging and contours. Both plans were generated with a maximum dose of 75 Gy.

Details about ZAP-X treatment planning are described by Adler et al. [4]. The CyberKnife treatment planning procedure for TN has been described in detail by Romanelli et al. [8].

## Results

The time to develop the plan was about the same for both systems, about five minutes each. The expected duration of treatment is 25 minutes for ZAP-X and 33 minutes for CyberKnife. ZAP-X planning used two isocenters (4-mm collimators) with inverse planning delivering 29,441 monitor units (MU) through 81 beams, with a conformity index of 1.66 and target coverage of 87%. CyberKnife planning used a 5-mm collimator with non-isocentric planning. While the same contour was used on both treatment planning systems, the planning was done independently of the contour. As a point of reference, Gamma Knife® (Elekta, Stockholm, Sweden) TN plans frequently utilize a single isocentric plan. In such instances, the coverage would be significantly less than 87%. The CyberKnife plan delivered 17,880 MU and 88 beams, with a conformity index of 1.33 and a target coverage of 98%.

**Figure 1:**
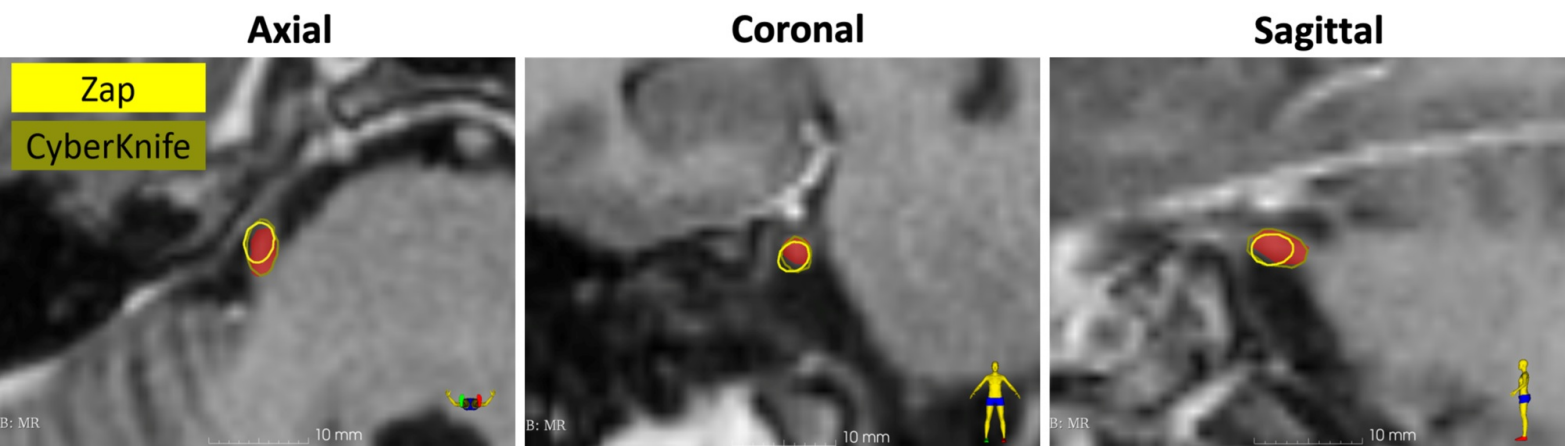
Comparison of the prescription isodose curves. Note that ZAP-X uses 70% and CyberKnife uses 80% as their prescription lines. ZAP-X: ZAP Surgical Systems, Inc., San Carlos, CA; CyberKnife: Accuray Incorporated, Sunnyvale, CA

**Figure 2:**
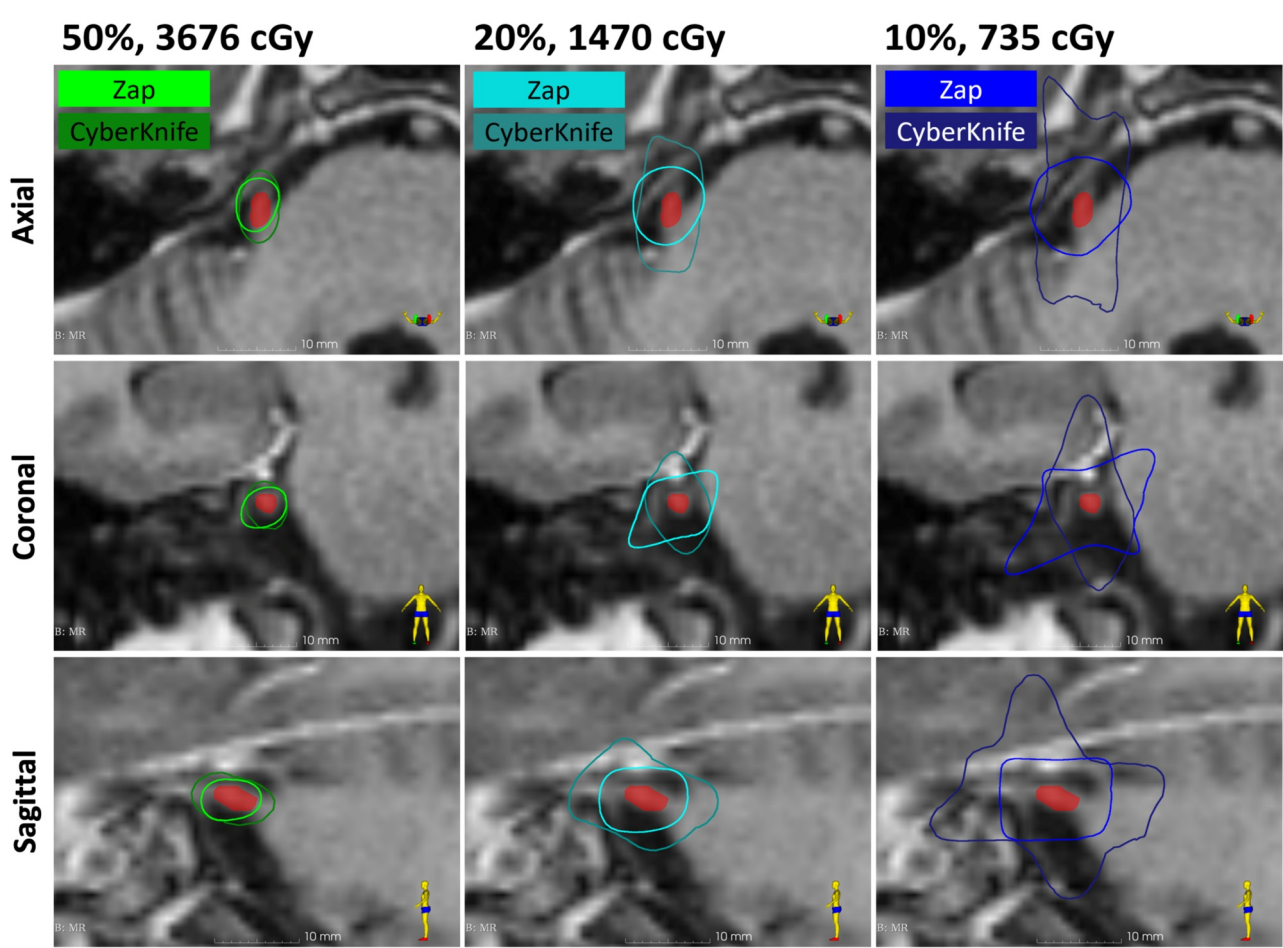
50%, 20%, and 10% isodose lines for ZAP-X and CyberKnife ZAP-X: ZAP Surgical Systems, Inc., San Carlos, CA; CyberKnife: Accuray Incorporated, Sunnyvale, CA

A detailed analysis revealed that the isodose volumes covered by ZAP-X and CyberKnife were similar at the 70% and 80% prescription isodose lines, respectively ( 0.02 cc for ZAP-X and 0.03 cc for CyberKnife), with 7500 cGy being the maximum dose (Dma)x for both plans (Figure 1). However, the isodose volumes were smaller for the 50%, 20%, and 10% isodoses for ZAP-X (Figure 2). The 20% and 10% isodose volumes were much smaller for ZAP-X, especially on the axial and sagittal planes. Specifically, for ZAP-X and CyberKnife, respectively, the 50% volumes were 0.12 cc and 0.18 cc, the 20% isodose volumes were 0.57 cc and 0.91 cc, and the 10% isodose volumes were 1.69 cc and 3.41 cc. Of special note, qualitative visual inspection by skilled practitioners of radiosurgery deemed both plans to be roughly comparable.

## Discussion

Radiosurgical treatment of TN is technically challenging. Sub-millimetric spatial accuracy is required to ensure the selected segment of the affected TN can be safely irradiated in a single treatment. A typical radiosurgical treatment lasts approximately 30 minutes and administers a radiation dose as high as those given over a month through conventional radiation therapy. The danger inherent to an inaccurate treatment is delivering dangerous doses to the brainstem and Gasserian ganglion, with potentially serious neurological consequences. Frame-based radiosurgery, either performed with Gamma Knife or LINAC-based SRS, has widely shown its ability to precisely target the trigeminal nerve, thus providing pain relief over time [5]. Frameless image-guided robotic radiosurgery with the CyberKnife has recently emerged as an equally accurate, safe, and effective treatment modality for TN, thus providing a less invasive non-medical treatment for this neurological disorder [7-8].

In this report, the differences in plans generated by the two teams are important to note. By using two isocenters with the ZAP-X, one would expect the coverage to be lower and conformity index to be higher; in contrast, the typical Gamma Knife trigeminal rhizotomy uses a single isocenter. Moreover, since ZAP-X uses a lower energy (3 MV) beam, the number of delivered monitor units (MUs) is, not surprisingly, higher.

Given slight differences in technology and approach to treatment planning, it was decided to not use the same prescription dose or prescription isodose curve. We recognize that with cancer treatments, it is more standard to reference such prescription (marginal) doses. However, to simplify comparisons in the current study, involving radiosurgical lesioning, the authors chose to keep Dmax constant for both devices and treatment planning systems.

ZAP-X is a self-shielded radiosurgical system requiring no vault [1-4]. The device has been developed to provide self-containment of the radiation originated by a 3 MV LINAC rotating around the target via dual yoked gimbal gantries. A thermoplastic mask holds the head of the patient in the desired position. Target localization is achieved by an integrated planar kilovolt (kV) imaging system providing 3D patient registration. Automatic realignment is performed prior to and throughout the treatment. The dose rate is 1500 MUs per minute with a Source-to-Axis Distance (SAD) of 45 cm. Beam collimation is provided by a revolving tungsten collimator with eight different sizes (4 mm to 25 mm). The system can change the collimator automatically during the treatment using a collimator wheel. Isocentric and non-isocentric planning can be performed. Dosimetric validation is given by a megavoltage (MV) image detector providing a real-time check of the administered dose.

The combination of a lower energy LINAC with a 4-mm collimator and the ability to develop both isocentric and non-isocentric planning allows ZAP-X to deliver a homogeneous dose to an extended segment of the TN, which proved safe and effective with the CyberKnife experience [7-8]. Further, the favorable penumbra of the ZAP-X 4-mm collimator relative to the 5-mm CyberKnife collimator (1.8 mm vs 2.1 mm) limits the exposure of critical structures such as the brainstem and the Gasserian ganglion [3,9]. Based on these few data points, ZAP-X may provide a dosimetric advantage for frameless image-guided TN SRS.

## Conclusions

Both qualitative visual inspection and more quantitative measurements suggest that the treatment of TN with ZAP-X is broadly comparable to that with CyberKnife. Nevertheless, a more detailed comparison shows that volumes receiving low doses (10%-20% isodose volumes) are smaller for ZAP-X plans than for CyberKnife plans; this observation could prove to be of potential clinical value. In summary, our investigation supports the use of ZAP-X for TN SRS.
